# Association Between 25(OH)Vitamin D, HbA1c and Albuminuria in Diabetes Mellitus: Data From a Population-Based Study (VIDAMAZON)

**DOI:** 10.3389/fendo.2021.723502

**Published:** 2021-10-07

**Authors:** João Soares Felício, Hana Andrade de Rider Britto, Pedro Celeira Cortez, Fabrício de Souza Resende, Manuela Nascimento de Lemos, Lorena Vilhena de Moraes, Vitória Teixeira de Aquino, Fernanda de Souza Parente, Natércia Neves Marques de Queiroz, João Felício Abrahão Neto, Angélica Leite de Alcântara, Wanderson Maia da Silva, Norberto Jorge Kzan de Souza Neto, Pedro Paulo Freire Piani, Ícaro José Araújo de Souza, Lilian de Souza D’Albuquerque Silva, Maria Clara Neres Iunes de Oliveira, Nivin Mazen Said, Gabriela Nascimento de Lemos, Franciane Trindade Cunha de Melo, Daniela Lopes Gomes, Ana Carolina Contente Braga de Souza, Melissa de Sá Oliveira dos Reis, Valéria Suênya Galvão Leal, Isabel Jane Campos Lobato, Karem Miléo Felício

**Affiliations:** Endocrinology Division, University Hospital João de Barros Barreto, Federal University of Pará, Belém, Brazil

**Keywords:** epidemiology, endocrinology, diabetes, vitamin D, HbA1c, albuminuria

## Abstract

**Background:**

The effect of glycemic control on diabetic kidney disease (DKD) is well known. Recent evidence has suggested that Vitamin D (VD) may have a nephroprotective effect in diabetes, but the relationship between VD, glycemic control, and albuminuria has yet to be clarified.

**Objective:**

Evaluate the relationship between 25-hydroxy-vitamin D [25(OH)D], HbA1c, and albuminuria in Diabetes Mellitus (DM).

**Patients and Methods:**

Cross-sectional study with 1576 individuals with DM who had 25(OH)D, HbA1c, and albuminuria levels measured. Patients with abnormal creatinine levels were excluded, in order to avoid interference on VD levels by impaired kidney function.

**Results:**

Patients with HbA1c ≥7% had lower 25(OH)D when compared to patients with HbA1c <7% (29.7 ± 10.2 *vs* 28.1 ± 9.9 ng/ml, p = 0.003) and 25(OH)D levels seems to predict 1.5% of HbA1c behavior. The 25(OH)D concentrations in patients with normoalbuminuria were higher than the levels observed in those with micro or macroalbuminuria (29.8 ± 9.0 *vs* 26.8 ± 8.6 and 25.1 ± 7.6, respectively, p = 0.001), patients who had 25(OH)D <20 ng/ml and 25(OH)D <30 ng/ml were at a higher risk of presenting albuminuria [OR = 2.8 (95% CI = 1.6 – 4.9), p<0.001, and OR = 2.1 (95% CI = 1.3 - 4.6), p<0.001, respectively]. In our regression model, albuminuria was influenced by HbA1c (r² = 0.076, p<0.00001) and 25(OH)D (r² = 0.018, p = 0.002) independently.

**Conclusion:**

Our study found an association between vitamin D levels, HbA1c and DKD. Additionally, our data suggest that the association between urinary albumin excretion and vitamin D levels is independent of glycemic control in patients with diabetes. Even though our patients presented normal creatinine levels, it is necessary further prospective studies to confirm if this association precedes or not the loss of renal function.

## Background

The effect of glycemic control on diabetic kidney disease (DKD) is well known. Large clinical trials, such as DCCT in type 1 Diabetes Mellitus (DM) and UKPDS in type 2 DM (1–3), clearly demonstrated the benefits of improving glycemic control on primary and secondary prevention of DKD. Recent evidence has suggested that Vitamin D (VD) may have a nephroprotective effect in DM (4, 5), and other studies have associated 25-hydroxy-vitamin D [25(OH)VD] levels with albuminuria (6, 7), but it is not a consensus (8).

Several mechanisms are suggested to explain the action of VD in reducing urinary albumin excretion in diabetic patients, including the suppression of renin transcription, proliferative and fibrotic effects, or a combination of these (9, 10). However, there are few evidence showing that VD deficiency or insufficiency in diabetic patients could be associated with microalbuminuria (11) and macroalbuminuria (3) and none of them was a population-based study.

The relationship between 25(OH)D levels and glycemic control also remains unclear. While cross-sectional studies have suggested a possible association (12, 13), others have not (14, 15). In addition, the attempts to supplement VD to improve glycemic control have also shown conflicting results (16, 17). Furthermore, a recently published meta-analysis found that VD supplementation may prevent type 2 diabetes mellitus (T2DM), showing the importance of this subject (18). Therefore, this study aims to evaluate the relationship between serum 25(OH)D levels, glycated hemoglobin (HbA1c), and albuminuria in a based-population study with diabetic patients.

## Methods

### Study Design and Data Collection

A cross-sectional study was performed, on a populational basis, to evaluate the association between the serum levels of 25(OH)D, HbA1c, and albuminuria in individuals with Diabetes Mellitus. This study was approved by University Hospital João de Barros Barreto ethics committee, CAEE number 66977717.8.0000.0017, conducted according to the Declaration of Helsinki and the standards of the National Health Council.

The present study used the large VIDAMAZON database, which includes data from over 30,000 individuals and has already provided evidence on other VD aspects (19). We included 1,576 diabetic patients who had their diagnosis confirmed by laboratory data and medical history, but we had no access to their medical records and our laboratory data did not contain antibodies related to type 1 DM and C-peptide. Inclusion criteria consisted of patients diagnosed with diabetes mellitus according to American Diabetes Association criteria (20). Patients with abnormal serum creatinine levels were excluded. Those with a previous history of acute or chronic kidney failure and chronic liver disease were excluded, as well as pregnant women and patients who supplemented vitamin D.

We included 1,576 subjects with type 1 and type 2 diabetes mellitus of both sexes and from different age groups. They had 25(OH)D serum levels, glycated hemoglobin, serum creatinine, fast glycemia, and albuminuria measured at a local laboratory service from August of 2017 to July of 2018. Albuminuria samples were available only in 496 patients. Information regarding BMI and living zone were also collected.

### Assay

Serum 25(OH)D was measured quantitatively by the following kit: DiaSorin LIAISON 25(OH)Vitamin D TOTAL chemiluminescence immunoassay (DiaSorin, Stillwater, MN, USA) (21). DiaSorin LIAISON is one of the methods to evaluate 25(OH)D tested by DEQAS (Vitamin D External Quality Assessment Scheme), the largest specialist external quality assessment (proficiency testing) scheme for the vitamin D metabolites 25(OH)D and a 1.25(OH)2D (22).

In data analysis, Vitamin D normal values were stated according to the Institute of Medicine (IOM) and Endocrine Society (ES) criteria. IOM established Vitamin D healthy serum values as ≥ 20 ng/dl (23, 24) and ES defines values ≥ 30ng/mL as sufficient, values between 20 and 30 ng/mL as insufficiency and values < 20 ng/mL as deficiency (25). Albuminuria was determined by turbidimetric immunoassay. The creatinine was determined through Jaffé technique (26, 27). Normoalbuminuria was defined as < 30mg/g creatinine, microalbuminuria ≥ 30 mg/g creatinine and < 300 mg/g creatinine, and macroalbuminuria as ≥ 300 mg/g creatinine. HbA1C was measured by ionic exchange high-performance liquid chromatography (HPLC) (28). The calculation of Glomerular Filtration Rate (GFR) was done by the Chronic Kidney Disease Epidemiology Collaboration equation (CKD-EPI) (29).

### Statistical Analysis

Variables with normal distribution were arranged in the form of a mean and standard deviation, while variables with non-normal distribution were arranged in the form of median and interquartile range. For the comparison of means, the Student’s-T test was used for variables with normal distribution and the Mann-Whitney test for variables with non-normal distribution. Simple linear regression and stepwise multiple regression analysis were also performed to evaluate the influence of 25(OH)VD levels in variables such as HbA1c and albuminuria. Simple linear regressions analysis was carried out to assess the influence of 25(OH)VD levels on HbA1c and albuminuria, using the Kolmogorov-Smirnov test to define variables normality. Additionally, a stepwise multiple regression model was developed, using 25(OH)VD and HbA1c as covariates to determine if these variables were independently associated with albuminuria. The ANOVA or Kruskal-Wallis tests were used to compare the variables between three subgroups according to normality. Albuminuria data ​​were presented in the form of a base logarithm 10.

The SPSS Statistics 22^®^ (IBM Corp.) and SigmaPlot 12.0 (Systat Software, Chicago, IL) programs were used for statistical analysis. A p-value less than 0.05 was considered significant.

## Results

Clinical and laboratory characteristics of all patients are presented in **Table 1**. According to Hb1Ac levels, 432 (27%) of patients presented HbAc1 levels < 7%, while 846 (54%) had their serum levels between 7 and 10% and 298 (19%) presented HbA1c > 10%.

**Table 1:** Patients’ clinical and laboratory characteristics.

Parameter	(N = 1576)
**Sex (F/M)**	988/588
**Age (years)**	62.2 ± 12
**BMI (Km/m²)**	29.5 ± 5
**Arterial Hypertension (yes %)**	430 (27%)
**HbA1c (%)**	8.3 ± 2
**25(OH)VD (ng/mL)**	28.5 ± 10
**Fasting Glucose (mg/dL)**	161 ± 60
**Creatinine (mg/dL)**	0.8 ± 0.2
**GFR (ml/min/1.73m^2^)**	89 ± 16

BMI, Body Mass Index; HbA1c, Glycated Hemoglobin; GFR, Glomerular filtration rate.

**Figure 1:**
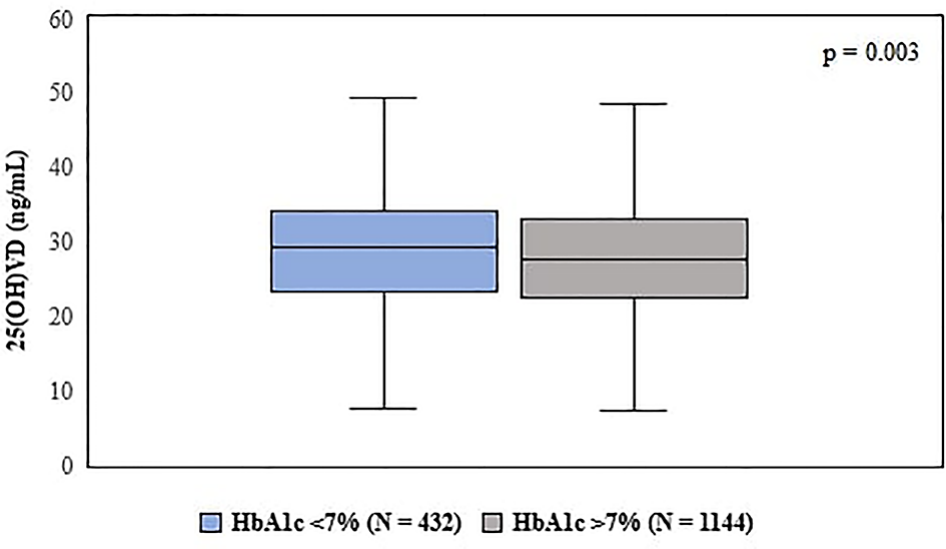
Comparison of vitamin D levels according to patient’s HbA1c range. Statistical test used: Mann–Whitney U test.

**Figure 2:**
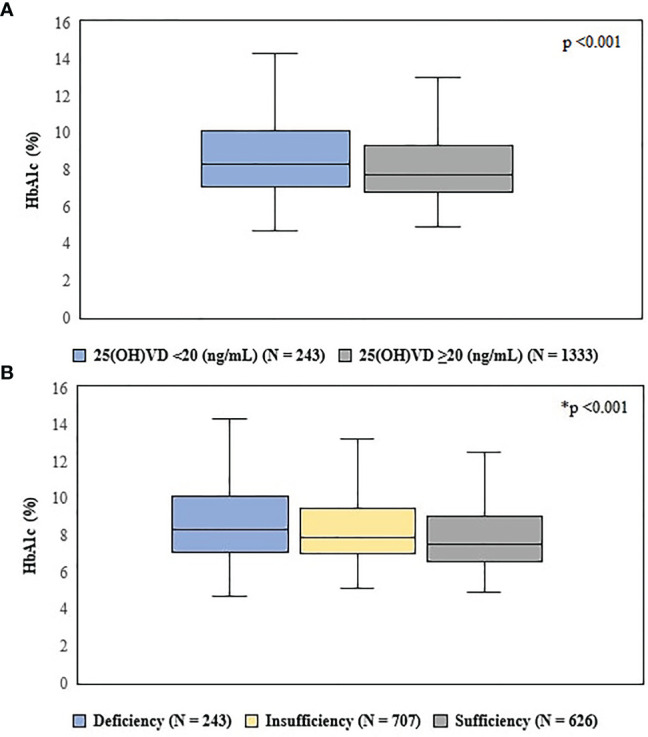
Comparison of HbA1c levels, according to Vitamin D status. **(A)** = defined by the 97.5 percentile, indicated by the Institute of Medicine. Statistical test used: Mann–Whitney U test. **(B)** = according to Endocrine Society criteria. Statistical test used: Kruskal-Wallis test. Deficiency = 25(OH)VD<20 ng/ml; Insufficiency = 25(OH)VD between 20 and 30 ng/ml; Sufficiency = 25(OH)VD ≥ 30 ng/ml. *p < 0.05 between all groups.

We observed that patients with lower HbA1c levels presented higher Vitamin D values. In our analysis, those patients who presented HbA1c >7% had significantly lower 25(OH)VD concentration when compared to patients who had HbA1c <7% (28 ± 10 *vs* 29.5 ± 10 ng/ml, p = 0.003) (**Figure 1**). Furthermore, using different cut-off points to normal VD levels, our data demonstrate that glycemic control assessed by HbA1c was better in patients with higher 25(OH)VD levels (**Figure 2**).

**Figure 3:**
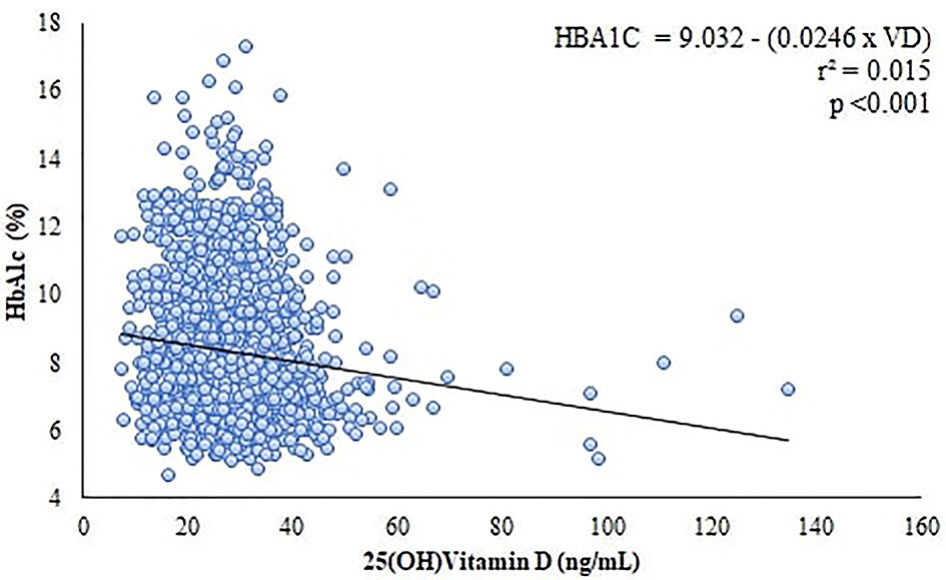
Correlation and linear regression between Vitamin D levels and HbA1c.

Our simple linear regression model using 25(OH)VD and HbA1c showed that an increase of 10 ng/dL in 25(OH)VD concentration was associated with a decrease of 0.25% in HbA1c. Besides that, 25(OH)VD levels seemed to be able to predict 1.5% of HbA1c behavior (**Figure 3**).

**Figure 4:**
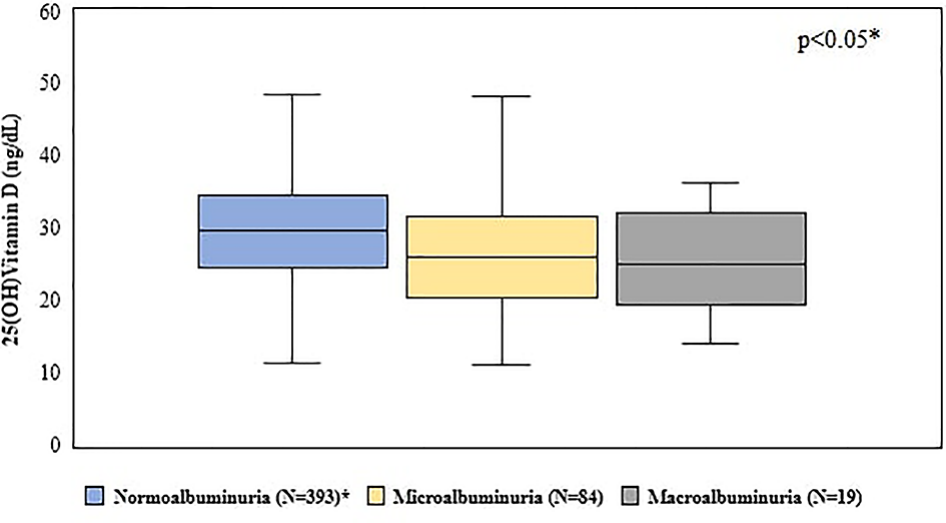
Comparison between vitamin D levels according to albuminuric stages. *p < 0.05 between normoalbuminuria *vs* microalbuminuria and *vs* macroalbuminuria. Statistical test used: Kruskal-Wallis test.

**Figure 5:**
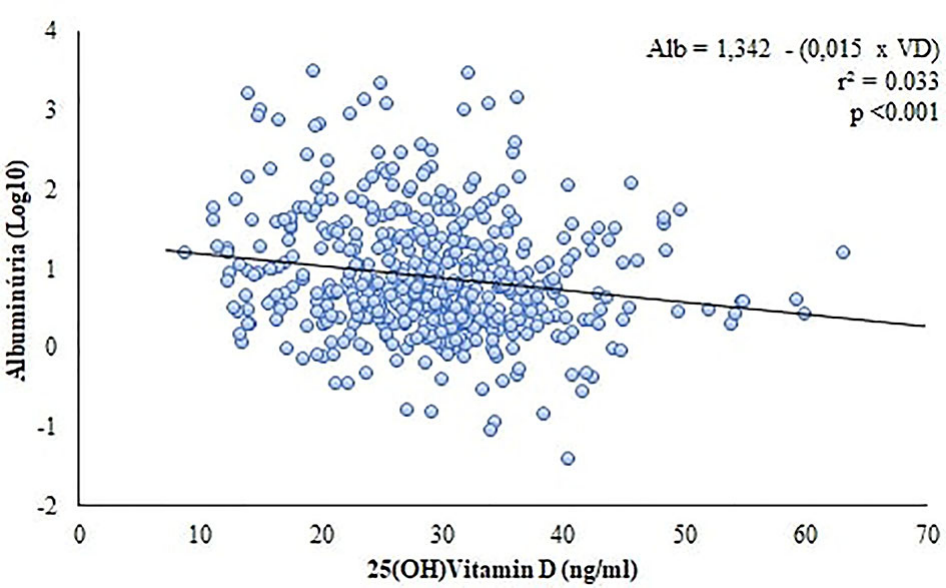
Linear regression between vitamin D levels and albuminuria.

We have found a relationship between albuminuria and VD levels. The 25(OH)VD concentration in patients with normoalbuminuria was higher than the levels observed in those with micro or macroalbuminuria (29.8 ± 9 *vs* 26.8 ± 8.6 and 25.1 ± 7.6, respectively) (**Figure 4**) and patients who had 25(OH)VD <20 ng/ml and 25(OH)VD <30 ng/ml were at a higher risk of presenting albuminuria [OR = 2.8 (95% CI = 1.6 – 4.9), p<0.001, and OR = 2.1 (95% CI = 1.3 - 4.6), p<0.001, respectively]. In addition, our simple linear regression model using albuminuria and VD demonstrated that 3% of albuminuria behavior could be explained by 25(OH)VD (**Figure 5**).

In a stepwise multiple regression model, albuminuria was found to be influenced by HbA1c (B = 0.253, r² = 0.076, p<0.00001) and 25(OH)VD (B = -0.137, r² = 0.018, p = 0.002) independently.

## Discussion

Our data demonstrated that patients who had good glycemic control had higher VD levels, with an increase of 10 ng/dL in VD concentration being associated with a decrease of 0.25% in HbA1c. Moreover, our data showed that 25(OH)D concentration ranges established by the Endocrine Society were able to predict better glycemic control evaluated by HbA1c, suggesting that levels > 30ng/dL could be associated with a VD maximum effect on glycemic control.

Cross-sectional studies conducted by Luo et al. (14) and Al-Showmer et al. (15) found no significant relationship between 25(OH)D levels and HbA1c. However, both studies were conducted with small samples (109 and 129 patients, respectively). In contrast, Salih et al. (13), studying 310 patients with T2DM, showed a high prevalence of hypovitaminosis D in those patients when compared to controls. In addition, Darraj et al. (12), in an analytical cross-sectional study with 309 T2DM patients in Saudi Arabia, has found that VD deficiency was an independent predictor for poor glycemic control after adjustment for hypertension, diabetes duration, and diabetic retinopathy and neuropathy. They have not evaluated DKD. The present study used the large VIDAMAZON database (19), being, as we are aware, the first population-based study to establish the association of higher VD levels with better glycemic control.

Our study demonstrated a clear association between higher VD levels and better glycemic control, showing the importance of studying this issue in detail. Nevertheless, it is still difficult to recommend VD supplementation to all prediabetics and diabetics patients. In fact, there is no consensus regarding the relationship between glycemic control and vitamin D supplementation, with studies pointing to opposite directions (16, 17, 30, 31). Alaidarous et al. (32), studying patients with T2DM have found that VD levels were affected by diabetes duration. A recently published meta-analysis found that VD supplementation may prevent T2DM (18), bringing VD reposition closer to our clinical practice.

One hypothesis that could explain our results is that VD could act in insulin resistance, and consequently decreasing glycemic values. The action of VD on insulin resistance has been documented in several studies (33–35). In a study with 126 healthy patients, the authors demonstrated a positive correlation of VD with insulin sensitivity and a negative effect with VD deficiency on beta-cell function (36). Vitamin D also acts directly on beta-cells facilitating insulin secretion from the binding of 1.25(OH)2D3 to its nuclear VD receptor and indirectly by regulating the flow of calcium in those cells (37, 38). Changes in the concentrations of this mineral can lead to peripheral resistance to insulin action, by reduction of signal transduction in glucose transport activity.

In our study, VD concentration in patients with normoalbuminuria was higher than in those with micro and macroalbuminuria. We also found that diabetic patients who had VD insufficiency and deficiency were at a higher risk of developing DKD.

In a cross-sectional analysis, Xie et al. (39) in 2019 studied a group of 351 T2DM patients with glomerular filtration rate (GFR) > 60 mL/min and found that low VD levels were independently associated with DKD. In a previous study, we also found a direct relationship between VD and albuminuria in type 1 diabetes mellitus (T1DM) patients with normal GFR, in which those with low VD levels had higher levels of albuminuria, and VD became lower as DKD stages worsened (6). The current study corroborates these findings in a population base. Although vitamin D supplementation might be useful as adjuvant therapy for albuminuria, this relationship is still uncertain, highlighting the need for further studies and with a larger sample size (40).

Our results agree with De Boer et al. (7), who found an association between patients with low 25(OH)D levels (< 20ng/mL) and higher risk of microalbuminuria. In addition, Verrotti et al. (41) studied VD levels in 22 patients with microalbuminuria, 24 patients with normoalbuminuria, and 24 healthy controls, in which the first group showed lower levels of 25(OH)D. In contrast, Joergensen et al. (8) analyzed 220 T1DM patients diagnosed from 1979 to 1984 and did not find an association between albuminuria and VD deficiency, but their results could have been affected by long duration storage of VD samples.

Vitamin D has an important role in DKD. It is well known that diabetic patients with DKD have urinary excretion of VD and VD binding protein, suggesting an impairment reuptake in proximal tubule cells (42). It has been recently described in the literature that VD seems to have a nephroprotective effect (4, 5, 43). One possible mechanism is an action as a renin-angiotensin system inhibitor (44). In addition, VD may act lowering oxidative stress by improving renal antioxidant capacity, preventing damage to podocytes by inhibiting hyperglycemia-induced apoptosis, promoting anti-inflammatory action, and improving endothelial function (9, 10, 45–47). Those findings are corroborated by our regression model, in which both HbA1c and VD were selected as independent variables influencing albuminuria.

Some studies with large samples evaluated the association between VD levels and DKD (48–50). Hong et al. (48), studying retrospectively 1392 patients with T2DM found an association between VD levels, albuminuria, and DKD. In parallel, Wang et al. (50), in an observational study evaluating 4033 patients, described those patients with low VD levels showed a stronger association between lead levels and urine albumin-to-creatinine ratio. Both studies included patients with impaired renal function. In our study, we described the association between VD levels, HbA1c, and albuminuria in patients with normal creatinine levels, suggesting that VD is linked to albuminuria before kidney function declines. Our data must be evaluated with caution because we were not capable to exclude subclinical forms of renal insufficiency.

The strengths of our work include a great number of patients in a populational study, excluding patients with evident impaired kidney function (high serum creatinine levels), in order to avoid its interference on VD metabolism. Our main limitation was the difficulty to access information about other factors that could interfere with VD levels (sun exposure, dietary information, and diabetes duration). The 1,576 patients with diabetes included in this work had their diagnosis confirmed by laboratory data and medical history, but we had no access to their medical records and our laboratory data did not contain antibodies related to T1DM and C-peptide. Therefore, our work did not differentiate diabetes types. In addition, we had a small number of patients with macroalbuminuria.

## Conclusion

Our study found an association between vitamin D levels, HbA1c and DKD. Additionally, our data suggest that the association between urinary albumin excretion and vitamin D levels is independent of glycemic control in patients with diabetes. Even though our patients presented normal creatinine levels and in consideration of the limited sensitivity and specificity of urinary albumin excretion to correctly identify incipient DKD, it is necessary further prospective studies to confirm if this association precedes or not the loss of renal function.

## Data Availability Statement

The original contributions presented in the study are included in the article/supplementary material. Further inquiries can be directed to the corresponding author.

## Ethics Statement

The studies involving human participants were reviewed and approved by University Hospital João de Barros Barreto ethics committee. Written informed consent from the participants’ legal guardian/next of kin was not required to participate in this study in accordance with the national legislation and the institutional requirements.

## Author Contributions

All persons who meet authorship criteria are listed as authors, and all authors certify that they have participated sufficiently in the work to take public responsibility for the content, including participation in the concept, design, analysis, writing, or revision of the manuscript. KF, JF, HB, and NQ took part in conception and design of study. AA, GN, PC, MR, VL, IL, DG, and AC were responsible for acquisition of data, while WS, ÍA, NMS, PF, NJS, and JA have done the analysis and interpretation of data. ML, JF, FS, LM, VA, FS, LS, FM, and MO have drafted the manuscript together. All authors contributed to the article and approved the submitted version.

## Conflict of Interest

The authors declare that the research was conducted in the absence of any commercial or financial relationships that could be construed as a potential conflict of interest.

## Publisher’s Note

All claims expressed in this article are solely those of the authors and do not necessarily represent those of their affiliated organizations, or those of the publisher, the editors and the reviewers. Any product that may be evaluated in this article, or claim that may be made by its manufacturer, is not guaranteed or endorsed by the publisher.

## Abbreviations

25(OH)D, 25-hydroxy-vitamin D; DKD, Diabetic Kidney Disease; DM, Diabetes Mellitus; GFR, Glomerular Filtration Rate; HbA1c, Glycated Hemoglobin; T1DM, Type 1 Diabetes Mellitus; T2DM, Type 2 Diabetes Mellitus; VD, Vitamin D.
